# Copy Number Alteration Profile Provides Additional Prognostic Value for Acute Lymphoblastic Leukemia Patients Treated on BFM Protocols

**DOI:** 10.3390/cancers13133289

**Published:** 2021-06-30

**Authors:** Mirella Ampatzidou, Lina Florentin, Vassilios Papadakis, Georgios Paterakis, Marianna Tzanoudaki, Dimitra Bouzarelou, Stefanos I. Papadhimitriou, Sophia Polychronopoulou

**Affiliations:** 1 Department of Pediatric Hematology-Oncology, “Aghia Sophia” Children’s Hospital, 11527 Athens, Greece; v.papadakis@paidon-agiasofia.gr (V.P.); s.polychronopoulou@paidon-agiasofia.gr (S.P.); 2 Alfa Laboratory Diagnostic Center, YGEIA Hospital, 11524 Athens, Greece; lflorentin@alab.gr (L.F.); dbouzarelou@alab.gr (D.B.); 3 Laboratory of Flow Cytometry, Department of Immunology, “G.Gennimatas” General Hospital, 11527 Athens, Greece; genpater@gmail.com; 4 Department of Immunology, “Aghia Sophia” Children’s Hospital, 11527 Athens, Greece; m.tzanoudaki@paidon-agiasofia.gr; 5 Laboratory of Hematology, Department of Molecular Cytogenetics, “G.Gennimatas” General Hospital, 11527 Athens, Greece; s.papadimitriou@gna-gennimatas.gr

**Keywords:** acute lymphoblastic leukemia, child, genetics, MLPA, copy number alterations (CNAs), risk stratification, minimal residual disease

## Abstract

**Simple Summary:**

Recent advances in genomic analyses of acute lymphoblastic leukemia (ALL) have identified novel prognostic markers associated with patient outcome. In this frame, copy number alterations (CNAs) are constantly gaining relevance as potential risk stratification markers. Herein, we present our data of a proposed CNA-profile risk-index applied on a Greek ALLIC-BFM cohort. The results of our study demonstrate that EFS for GR(good-risk)-CNA-profile patients was 96.0% versus 57.6% of PR(poor-risk)-CNA-profile ones (*p* < 0.001) in the whole cohort. EFS within the IR-group for the GR-CNA vs. PR-CNA subgroups was 100.0% vs. 60.0% (*p* < 0.001), and within the HR-group, 88.2% vs. 55.6% (*p* = 0.047), respectively. The above results indicate that the application of the proposed CNA-profile classifier is feasible in BFM-based protocols, adding prognostic value to the existing prognostic markers and successfully stratifying patients within prognostic subgroups. This novel genomic risk index can be incorporated in future risk-stratification algorithms, further refining MRD-based stratification and possibly reassigning optimal treatment strategies.

**Abstract:**

We present our data of a novel proposed CNA-profile risk-index, applied on a Greek ALLIC-BFM-treated cohort, aiming at further refining genomic risk-stratification. Eighty-five of 227 consecutively treated ALL patients were analyzed for the copy-number-status of eight genes (IKZF1/CDKN2A/2B/PAR1/BTG1/EBF1/PAX5/ETV6/RB1). Using the MLPA-assay, patients were stratified as: (1) Good-risk(GR)-CNA-profile (*n* = 51), with no deletion of IKZF1/CDKN2A/B/PAR1/BTG1/EBF1/PAX5/ETV6/RB1 or isolated deletions of ETV6/PAX5/BTG1 or ETV6 deletions with a single additional deletion of BTG1/PAX5/CDKN2A/B. (2) Poor-risk(PR)-CNA-profile (*n* = 34), with any deletion of ΙΚΖF1/PAR1/EBF1/RB1 or any other CNA. With a median follow-up time of 49.9 months, EFS for GR-CNA-profile and PR-CNA-profile patients was 96.0% vs. 57.6% (*p* < 0.001). For IR-group and HR-group patients, EFS for the GR-CNA/PR-CNA subgroups was 100.0% vs. 60.0% (*p* < 0.001) and 88.2% vs. 55.6% (*p* = 0.047), respectively. Among FC-MRD_d15_ + patients (MRD_d15_ ≥ 10^−4^), EFS rates were 95.3% vs. 51.7% for GR-CNA/PR-CNA subjects (*p* < 0.001). Similarly, among FC-MRD_d33_ + patients (MRD_d33_ ≥ 10^−4^), EFS was 92.9% vs. 27.3% (*p* < 0.001) and for patients FC-MRD_d33_ − (MRD_d33_ < 10^−4^), EFS was 97.2% vs. 72.7% (*p* = 0.004), for GR-CNA/PR-CNA patients, respectively. In a multivariate analysis, the CNA-profile was the most important outcome predictor. In conclusion, the CNA-profile can establish a new genomic risk-index, identifying a distinct subgroup with increased relapse risk among the IR-group, as well as a subgroup of patients with superior prognosis among HR-patients. The CNA-profile is feasible in BFM-based protocols, further refining MRD-based risk-stratification.

## 1. Introduction

The survival rates in pediatric acute lymphoblastic leukemia (ALL) have improved significantly during the past decades, with more than 80% of patients achieving remission and long-term cure. This has been accomplished mainly by the refinement of risk stratification systems, the integration of MRD in current risk-adjusted therapy and the evolution of genome-wide technologies in exploring the underlying biology [1,2,3,4,5,6,7,8,9,10,11,12]. Nevertheless, the prevention of relapse and therapy-related toxicities present major challenges. In modern BFM-based stratification, the intermediate risk (IR) group represents the main patient group where most relapses come from, while treatment-related toxicities further challenge the high-risk (HR) patient group [13,14].

Triggered by these clinical needs, ALL blast genomic analyses aim to identify novel prognostic markers related to patient outcome [13,15,16,17,18,19,20,21,22,23,24,25,26,27]. Within this frame, individual copy number alterations (CNAs) involving deletions, duplications or amplifications of genes implicated in B-cell differentiation, cell cycle regulation, proliferation and transcription are constantly gaining relevance as potential risk stratification markers [15,16,17,18,19,20,21,22,23,24,25,26,27]. Additionally, the identification of commonly affected genes in childhood ALL has inspired the development of combined risk classifiers based on copy number status, such as the IKZF1^plus^ entity [14] and the United Kingdom (UK) ALL-CNA classifiers [25,26], in an effort to further improve patient outcome prediction. To date, however, most of the proposed genomic classifiers are not used for tailoring treatment decisions and their incorporation into risk-adjusted treatment protocols is yet to be defined. Furthermore, the disputable independent prognostic significance of such genomic markers in the context of MRD dependency remains an important challenge in patient stratification, posing questions for the feasibility of incorporating genomic classifiers in routine diagnostics and treatment [14,15,22,25].

Herein, we present a Greek ALLIC/BFM treated patient cohort for which a two-tier CNA-profile risk index has been applied, aiming at a novel genomic risk stratification and identification of distinct subgroups with different prognosis. The scope of the current study is to demonstrate: (1) the feasibility and robustness of the proposed CNA-classifier in BFM-based ALL treatment protocols, (2) the additive prognostic value of the CNA risk index to the established, used stratification markers and (3) the integration with MRD in predicting outcome and survival.

## 2. Materials and Methods

### 2.1. Patients

During the years 2000–2020, 227 ALL patients (137 males/90 females, median age 5.0 years (range 0.2–17.5)) were consecutively diagnosed and homogeneously treated according to BFM-based protocols in a single center, the Department of Pediatric Hematology–Oncology (T.A.O.) of Aghia Sophia Children’s Hospital in Athens, Greece. The diagnosis of B-cell or T cell precursor origin was established according to conventional FAB and immunophenotypic criteria. A total of 201/227 patients (88.5%) were diagnosed with B-cell precursor ALL and 26/227 patients (11.5%) as T-cell precursor ALL.

### 2.2. Diagnosis; Morphologic, Molecular and Cytogenetic Testing

All patients were investigated by morphology of bone marrow (BM) smears, histochemistry, immunophenotyping, conventional cytogenetics (G-banding), FISH and RT-PCR for the presence of the common ALL translocations.

### 2.3. Flow Cytometry (FC)

BM samples were investigated for leukemia-associated immunophenotypes and were assessed by flow-cytometry (FC) using 3–5-color antibody combinations, adapted to published AIEOP-BFM Consensus Guidelines 2016 for Flow Cytometric Immunophenotyping of Pediatric ALL [28]. Follow-up samples for minimal residual disease (MRD) study were collected from BM at days 15, 33, 78, week 22–24 before initiation, as well as at the end of maintenance therapy. All high-risk (HR) patients were also evaluated before each HR block. MRD was detected by flow cytometry, initially using 5 colors and, since 2019, 9 and 10 colors, for B-ALL and T-ALL, respectively. Sample analysis was performed with FC-500 and NAVIOS (Beckman-Coulter, Miami, FL, USA) flow cytometers, using CXP-Analysis or Kaluza (versions 1.3 and 2.1) software. For MRD detection, a minimum of 500,000 events were collected with count extrapolation up to 3,600,000 events if needed. Sensitivity of 0.1–0.01% was achieved in most cases, with a minimum of 20 events acquisition in the MRD gate [1,3,5,6].

### 2.4. G-Banding, FISH and RT-PCR

Bone marrow cells were cultured for 24, 48 and 72 h prior to G-Banding. A 300-banding resolution technique (300 bands per haploid set-300 bphs) was applied. FISH evaluation using commercial probe sets was performed in non-cultured cells for the detection of *ETV6-RUNX1, TCF3-PBX1, BCR-ABL1* fusion genes, *KMT2A* gene rearrangements as well as *ETV6, RUNX1, CDKN2A/2B* and other gene duplications, deletions or amplifications. Ficoll-Hypaque-purified BM samples (Sigma-Aldrich, Saint-Louis, MI, USA and Merck, Darmstadt, Germany) were studied by RT-PCR for the presence of the common translocations *ETV6-RUNX1, TCF3-PBX1, BCR-ABL1* and *KMT2A-AFF1*.

### 2.5. MLPA (Multiple Ligation Probe Amplification)

MLPA (multiple ligation probe amplification) was applied using the SALSA-MLPA P335 kit (MRC Holland, Amsterdam, The Netherlands). Among the 227 ALL patients consecutively treated in our department (49 SR/118 IR/60 HR), BM samples from 85 non-selected IR and HR patients were MLPA analyzed (retrospective: 45, prospective and consecutively diagnosed since 2015: 40), evaluating the copy number status detection of 8 genes: *IKZF1*, *CDKN2A/2B*, *PAR1*, *BTG1*, *EBF1*, *PAX5*, *ETV6*, *RB1*. The Salsa-MLPA-P335-Kit has been used according to manufacturer’s instructions [23,26]. Standard risk (SR) patients (*n* = 49) were not included in BM MLPA analysis.

### 2.6. Conventional Risk Stratification, Therapy Groups and Treatment Protocol

All patients were treated according to AIEOP-BFM-ALL-based protocols (BFM 1995/2000 and ALLIC-BFM 2009) [4,29,30]. Initial risk stratification was conducted according to protocol criteria [1,29,30]. All patients were stratified as good or poor prednisone responders (GPR or PPR) according to peripheral blood (PB) smears on day 8 of remission-induction therapy (absolute blast count < or ≥ 1000/μL).

Non-T ALL patients with WBC < 20,000/μL at diagnosis and age ≥ 1 to < 6 years that lacked high risk criteria and had an FC-MRD load on day 15 of < 0.1% when treated on ALLIC-BFM 2009 protocol were characterized as SR patients according to protocol stratification. The high-risk group included patients with any of the following: detection of KMT2A/AFF1; detection of BCR/ABL1; poor prednisone response on day +8; inability to achieve complete remission (CR) on day +33; hypodiploidy and FC-MRD ≥ 10% on day 15 for patients treated on ALLIC-BFM 2009 protocol. All other patients were allocated to the intermediate risk (IR) group, by protocol stratification.

The remission induction, consolidation and reinduction therapy have been applied according to the BFM-backbone, as previously described [1,3,5,6,12].

### 2.7. Copy Number Alterations (CNA)-Profile Risk Stratification

Based on copy number alterations (CNA) profile, the 85 patients evaluated by MLPA were further stratified in 2 distinct CNA risk groups according to the following criteria:

(1) Good risk (GR) CNA profile with:
  a. no deletion of *IKZF1, CDKN2A/B, PAR1, BTG1, EBF1, PAX5, ETV6, RB1* or
  b. isolated deletions of *ETV6, PAX5, BTG1* or
  c. *ETV6* deletions with a single additional deletion of *BTG1, PAX5 or CDKN2A/B*.
(2) Poor risk (PR) CNA profile with:
  a. any deletion of *IKZF1, PAR1, EBF1, RB1* or
  b. any other CΝA-profile not mentioned above.

Gene amplifications in the context of documented hyperdiploidy were not included in the CNA stratification algorithm.

### 2.8. Statistical Analysis

Event-free survival (EFS) and overall survival (OS) estimates were obtained using the Kaplan–Meier method and standard errors of the estimates were calculated using Greenwood’s formula. Time to relapse was calculated as the time from diagnosis to first relapse, while time to event was estimated as the time from diagnosis to the first adverse event (relapse, refractory disease, secondary malignancy or death). Patients were censored at the time of last follow-up. OS was defined as the time from diagnosis to death from any cause and patients were censored at the time of last follow-up. The log-rank test was used for comparison of survival curves between various groups. Multivariate analysis was conducted and prognostic factors for EFS and OS were identified using Cox proportional hazards regression model. The significance of covariate or factor effects was tested using Wald tests. Associations between categorical variables were tested using the X^2^ test. All tests were conducted at a significance level of 5% (*p* values ≤ 0.05 were considered statistically significant).

## 3. Results

### 3.1. Patient Characteristics and Conventional Risk Stratification

Among the 227 ALL patients consecutively treated in our department, during the years 2000–2020 (median follow-up time 113.9 months), 85 patients were checked by MLPA (Salsa-MLPA-P335-Kit), for the evaluation of the copy number status of 8 genes: *IKZF1*, *CDKN2A/2B*, *PAR1*, *BTG1*, *EBF1*, *PAX5*, *ETV6*, *RB1*.

Forty-eight/85 patients were male (56.5%) and 37/85 female (43.5%), with a median age at diagnosis of 5.6 years (range 0.2–16.7 years). Seventy seven of 85 patients (90.6%) were diagnosed with B-cell precursor ALL and 8/85 patients (9.4%) with T-cell precursor ALL. Median ranges for WBC, Hb and PLTs were 10,440/μL, 9.0 g/dL and 87,000/μL, respectively.

Conventional cytogenetic and molecular evaluation revealed *ETV6/RUNX1* translocation in 19/85 patients (22.3%), *KMT2A* gene rearrangements in 3/85 children (3.5%), *BCR/ABL1* translocation in 1/85 patient (1.2%) and 1/85 patient (1.2%) positive for the *TCF3-PBX1* aberration. Hyperdiploidy, defined by the number of chromosomes (>50) or DNA index (≥1.16) was detected in 18/85 patients (21.2%). There was no hypodiploidy or *TCF3-HLF* translocation detected in this patient cohort.

According to conventional protocol stratification criteria, 48/85 children (56.5%) were treated in the IR Arm and 37/85 patients (43.5%) in the HR Arm.

Patients’ baseline demographic, clinical, immunophenotypic, genetic and treatment response (MRD) features are presented in Table 1.

### 3.2. MLPA Results and CNAs

Using the MLPA assay for genomic screening, CNAs were detected in 46/85 patients (54.1%), with 34.8% of the cases (*n* = 16) harboring combined CNAs (≥2). The most common CNAs detected, sole or combined, were *CDKN2A/2B* deletion (41.4%, *n* = 17), *IKZF1* deletion (26.1%, *n* = 12), *ETV6* deletion (26.1%, *n* = 12) and *PAX5* gene deletion (13.0%, *n* = 6). Among T-ALL patients, *CDKN2A/2B* and *PAR1* were the only genes affected, with deletions present in 50% of the cases (4/8).

Among IR patients, by conventional protocol criteria (*n* = 48), CNAs were evaluated in 52.1% of the cases (*n* = 25). The most frequent CNAs detected, sole or combined, were *ETV6* deletion (20.8%, *n* = 10), *CDKN2A/2B* deletion (20.8%, *n* = 10) and *PAX5* deletion (10.4%).

Among HR patients, by conventional protocol criteria (*n* = 37), 59.4% of the cases presented with CNAs (*n* = 22), with *IKZF1* deletion (45.4%, *n* = 10) and *CDKN2A/2B* deletion (31.8%, *n* = 7) representing the most frequent CNAs detected.

**Figure 1:**
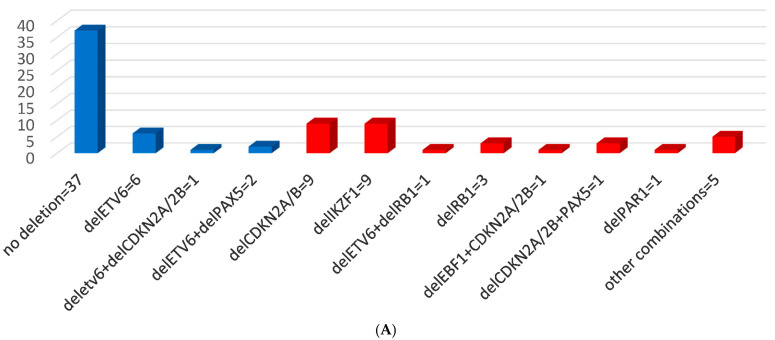

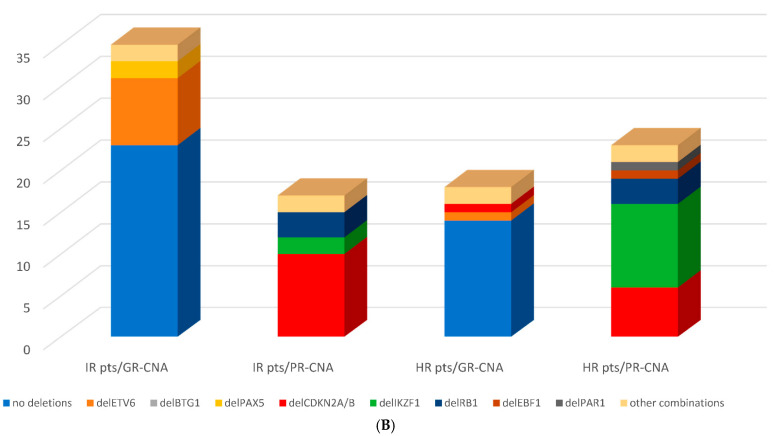
MLPA results and CNAs detected upon diagnosis. (**A**) MLPA results and CNAs in the whole cohort. Good risk (GR)-CNAs are displayed in blue color and poor risk (PR)-CNAs are shown in red. Other combinations include delCDKN2A/2B+delBTG1, delCDKN2A/2B+delRB1, delIKZF1+delCDKN2A/2B, delIKZF1+delCDKN2A/2B+delPAX5, delIKZF1+delETV6. Del = dele.

MLPA and CNAs results upon diagnosis are shown in Figure 1.

### 3.3. Genomic Risk Stratification—Detected CNA Profiles within Conventional Risk Groups

Applying the CNA profile stratification in the cohort of the 85 MLPA evaluated patients, 51/85 patients (60.0%) were classified as good risk CNA (GR-CNA)-profile and 34/85 patients (40.0%) were classified in poor risk CNA (PR-CNA)-profile subgroup.

Details regarding the detected CNA profiles within the conventional risk groups are presented in Table 2.

### 3.4. Outcome, Relapses and Survival Rates by CNA Profile

Analyzing the whole 85 patient cohort, overall survival (OS) and event-free survival (EFS) were 87.1% and 78.8%, respectively (median follow-up time of 49.9 months). For the established IR-group, by protocol stratification, EFS was 87.5% while the corresponding percentage for the HR-group was 67.6% (*p* < 0.001).

In the whole cohort evaluated by the MLPA assay, the genes associated with greater relapse probability were *CDKN2A/2B*, *RB1* and *IKZF1,* with relapse rates of 41.2%, 25% and 16.7%, in case of corresponding deletions. Isolated *ETV6* or *PAX5* gene deletions correlated with no relapse occurrence.

Applying the CNA profile algorithm and stratifying patients in two genomic CNA risk groups, OS and EFS for GR-CNA-profile patients were 96.0%/96.0% vs. 78.8%/57.6% for PR-CNA-profile patients (*p* = 0.015 for OS and *p* < 0.001 for EFS), in the whole cohort. GR-CNA-profile patients had a relapse rate of only 2.0%, compared to the PR-CNA-profile subgroup, in which the relapse rate was 38.2%.

Within the established IR-group (48/85), EFS was 100.0% for IR/GR-CNA-profile patients while IR/PR-CNA-profile subjects presented with EFS rates of only 60.0% (*p* < 0.001). The relapse rate was 0.0% vs. 33.3% for the IR/GR-CNA-profile compared to the IR/PR-CNA-profile subgroup.

Within the established HR-group (37/85), EFS rates accounted for 88.2% vs. 55.6% (*p* = 0.047) for the HR/GR-CNA-profile and HR/PR-CNA-profile subgroups, respectively. HR/GR-CNA-profile patients presented with low relapse rate (5.6%), while HR/PR-CNA-profile patients were associated with increased relapse rate of 42.1%.

Outcome of all patients stratified by CNA-profile are shown in Table 2.

**Figure 2:**
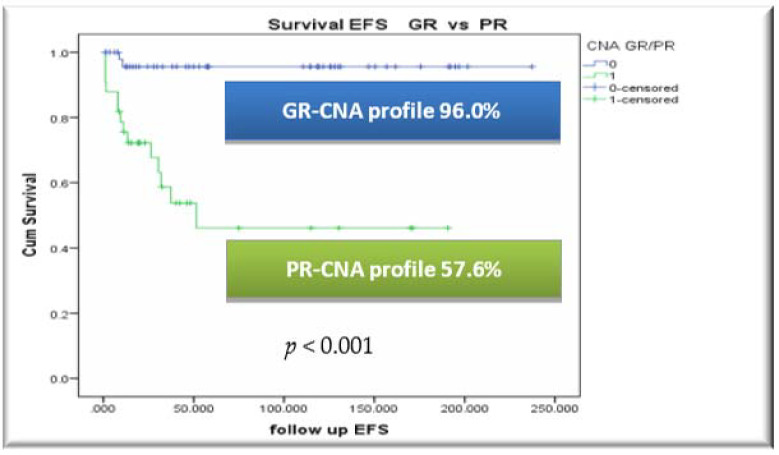

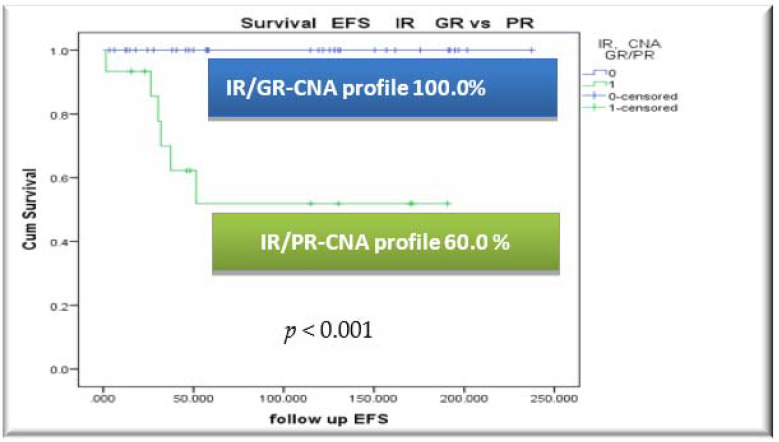

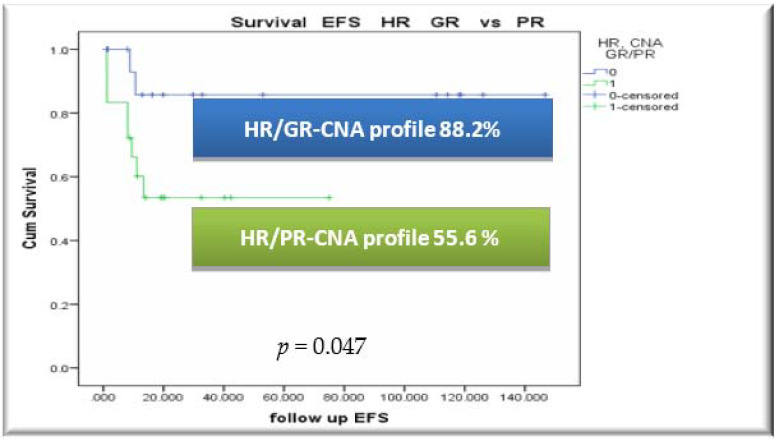
Survival rates by CNA risk index and EFS by CNA-profile within conventional protocol risk groups. (**A**) EFS by CNA profile in the whole patient cohort, (**B**) EFS by CNA profile within the intermediate risk (IR) group cohort, (**C**) EFS by CNA profile within the high risk (HR) group cohort.

Survival rates by CNA risk index and EFS by CNA-profile within conventional protocol risk groups, are presented in Figure 2.

### 3.5. CNA Profile and MRD Integration

Among FC-MRD_d15_+patients (MRD_d15_ ≥ 10^−4^), EFS rates were 95.3% vs. 51.7% for GR-CNA- and PR-CNA-profile subjects (*p* < 0.001), with corresponding relapse rates of 2.3% vs. 40.0%, respectively. No relapses occurred within the FC-MRD_d15_- subgroup (MRD_d15_ < 10^−4^).

Among FC-MRD_d33_+ patients (MRD_d33_ ≥ 10^−4^), EFS was 92.9% vs. 27.3% for GR-CNA and PR-CNA subgroups (*p* < 0.001). The relapse rate within the FC-MRD_d33_+ cohort was 0.0% for GR-CNA patients, compared to 54.5% for patients allocated to the PR-CNA-profile subgroup.

At the end of induction, patients with no detectable disease and FC-MRD_d33_ − (MRD_d33_ < 10^−4^) had EFS 97.2% if in the GR-CNA subgroup vs. 72.7% if in the PR-CNA subgroup (*p* = 0.004), with relapse rates of 2.8% vs. 22.7%, respectively (*p* < 0.001).

In an attempt to define the interaction modification between CNA profile and MRD, multivariate analysis was conducted and Cox regression analysis for EFS and OS was performed with the following covariables: protocol risk group, CNA profile and FC-MRDd33 status. The CNA profile was the most important prognostic factor for relapse, yielding a hazard ratio of 20.2 (95% confidence interval: 4.2–96.3, *p* < 0.001). Positive FC-MRDd33 status at the end of induction was also prognostic for relapse, with a hazard ratio of 8.5 (95% confidence interval: 1.9–35.9, *p* = 0.004). Regarding OS, the level for the CNA profile was the most important prognostic factor for survival, yielding a hazard ratio of 15.3 (95% confidence interval: 3.3–70.7, *p* < 0.001), with positive FC-MRDd33 status also retaining prognostic significance for survival, with a hazard ratio of 5.0 (95% confidence interval: 1.0–24.3, *p* = 0.044).

**Figure 3:**
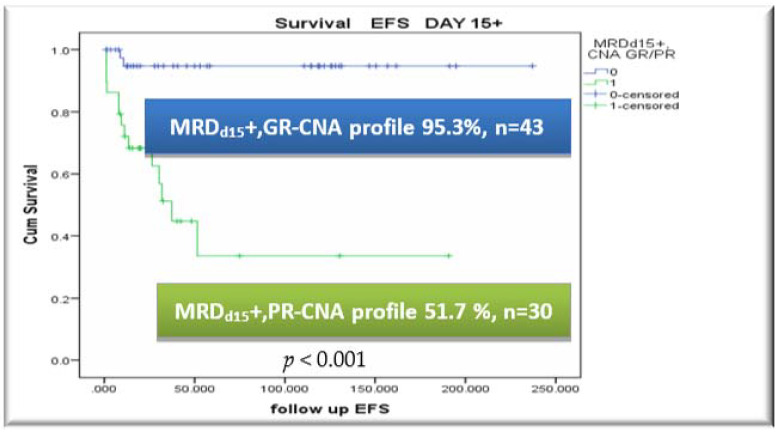

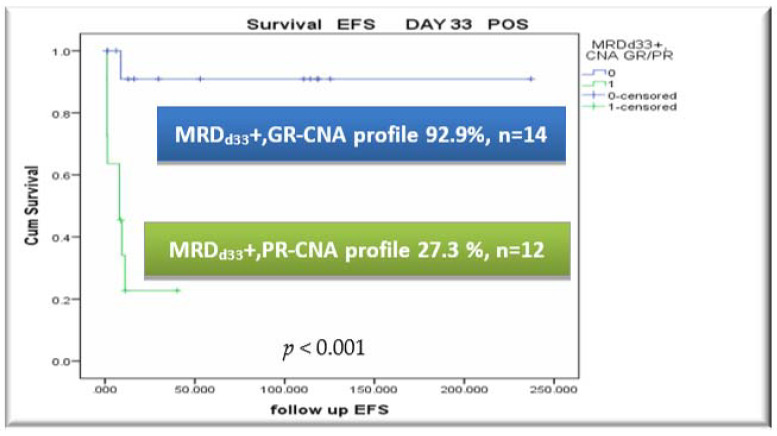

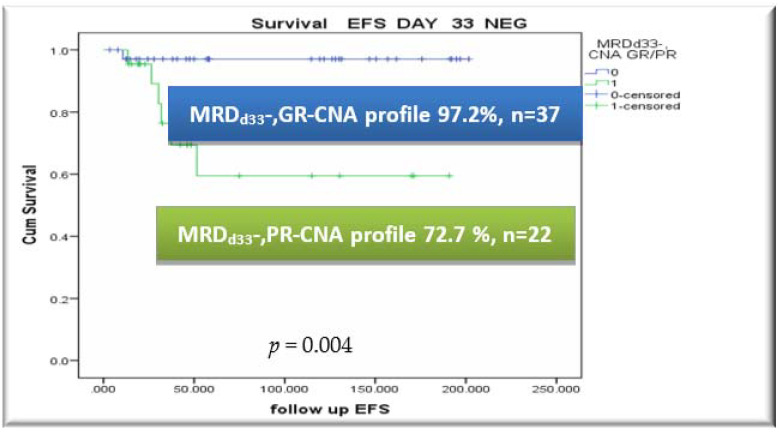

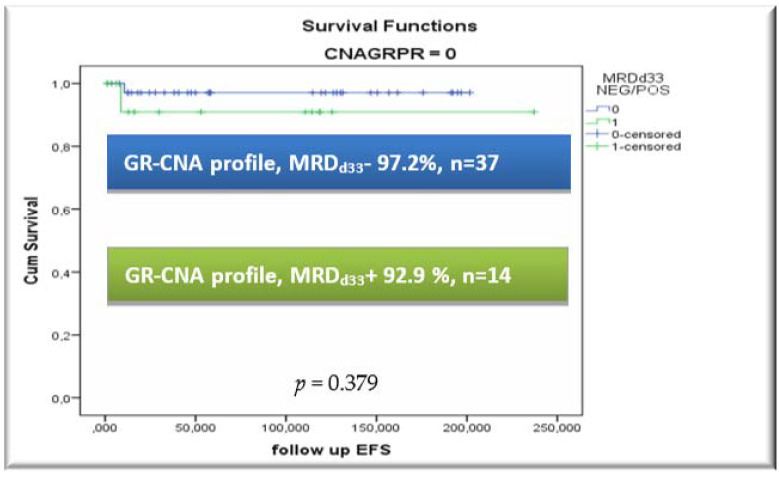

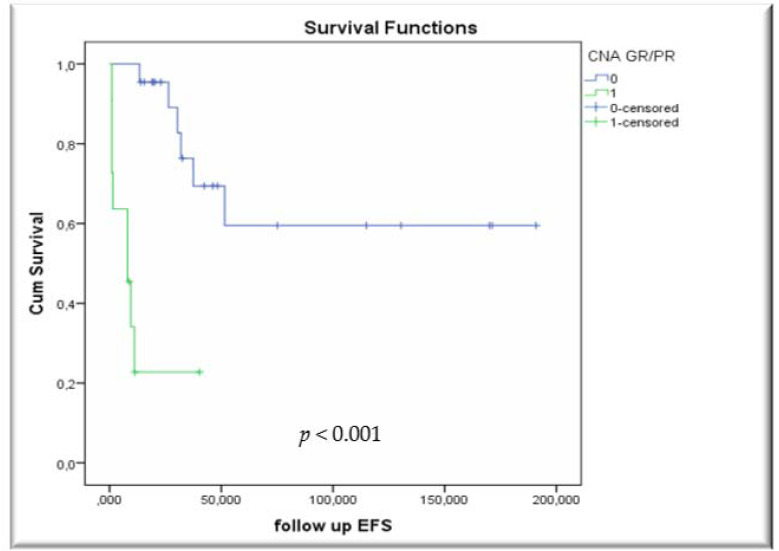
Survival rates by CNA risk index, integration with FC-MRD on days +15 and +33 and further stratification within FC-MRD positive and negative subgroups. (**A**) EFS by CNA profile among FC-MRDd15+ patients, (**B**) EFS by CNA profile among FC-MRDd33+ patients, (**C**) EFS by CNA profile among FC-MRDd33- patients, (**D**) EFS by MRDd33 status among GR-CNA patients, (**E**) EFS by MRDd33 status among PR-CNA patients.

Survival rates by CNA risk index, with integration of FC-MRD results on days +15 and +33 of induction treatment and further stratification within FC-MRD positive and negative subgroups, are presented in Figure 3A–C. Additionally, the prognostic value of FC-MRDd33 by CNA Profile is shown in Figure 3D,E.

## 4. Discussion

Recent insights into the underlying ALL biology are constantly gaining relevance in the prognostic classification of ALL during the past decade, mainly due to advances in genome-wide technologies [19,31,32]. Genomic assessments have identified numerous novel copy-number alterations (CNAs) that typically affect genes involved in lymphoid differentiation, proliferation, cell cycle regulation and transcription [25]. In contrast to cytogenetic chromosomal translocations, which are commonly initiating events, these CNAs are usually cooperating genomic aberrations that correlate with specific genomic subtypes and influence the ultimate patient outcome [23,24,25]. Nevertheless, one major limitation in assessing the prognostic relevance of individual CNAs is the fact that many cases harbor more than one deletion while other patients may have none. Therefore, alternative approaches have been attempted, with the integration of combined CNA profiles and classifiers, into the existing established risk group stratification [32].

In this study, utilizing experience from previously suggested genomic risk algorithms in various settings, we have tried to demonstrate the feasibility of a novel proposed CNA-classifier in BFM-based protocols, as well as to provide evidence on the additive prognostic value of this CNA risk index to all established stratification markers, including MRD, in predicting outcome and survival.

In our cohort, using the MLPA assay for genomic screening of isolated CNAs, the gene deletions associated with greater relapse probability were *CDKN2A/2B*, *RB1* and *IKZF1*, with relapse rates of 41.2%, 25% and 16.7%, respectively. Isolated *ETV6* or *PAX5* gene deletions correlated with no relapse occurrence. These findings are in concordance with large multi-institutional studies that suggest inferior survival rates in patients with *CDKN2A/2B, RB1* and *IKZF1* deletions [32,33,34,35,36,37,38,39,40,41,42,43,44]. The biallelic loss of the *CDKN2A/2B* tumor suppressor genes has long been proposed as an adverse prognostic marker, with other studies disputing the independent prognostic significance in case of heterozygosity and coexisting aberrations [31,32,40,41,42,43,44,45]. Similarly, the *IKZF1* gene deletion has been proposed as a genomic marker mediating drug resistance, conferring poor prognosis in various settings [32,33,34,35,36,37,38]. Nevertheless, despite the initial strong indications suggesting adverse prognosis of the *IKZF1* deleted subgroup, results from other study groups have emerged, including the AIEOP-BFM, questioning the independent prognostic significance in the absence of specific coexisting abnormalities and detectable MRD [14,36,37,45].

Taking into account all the above limitations and in an effort to overcome the problem of disputable independent prognostic significance of specific CNAs when evaluated alone, the development of combined CNA risk classifiers has evolved and is also being evaluated in the study presented. In the meantime, the AIEOP-BFM study group has introduced the IKZF1^plus^ entity [14,45] as the most important genomic risk classifier in predicting relapse. Although the IKZF1^plus^ subgroup has been identified as a poor prognostic marker [14,45], Stanulla et al. [14] reported that, among patients with no measurable MRD after induction, treatment outcome was not negatively affected by the presence of IKZF1^plus^ characteristics. Therefore, a major disadvantage was indicated, that of the MRD dependency in the era of modern MRD-adjusted protocols. Thus, the IKZF1^plus^ entity has a major limitation: it is MRD-dependent, identifying a small subgroup of poor risk patients and only among the MRD-positive patients at the end of induction treatment [14,15,26].

On the other side, Moorman et al. [25], since 2014, have retrospectively analyzed genetic data from 1500 patients to develop an integrative risk stratification algorithm, based on CNA and cytogenetic data and stratifying patients into groups with good- and poor-risk genetic alterations according to their integrated profile.

In concordance with the above, one of the most important results reported in our study, with the application of the CNA profile algorithm, was the statistically significant difference in survival outcomes: EFS for GR-CNA-profile patients was 96.0% vs. 57.6% for PR-CNA-profile patients (*p* < 0.001). GR-CNA-profile patients presented with a lower relapse rate of only 2.0%, compared to the PR-CNA-profile subgroup, in which the corresponding relapse rate was 38.2%, respectively. Thus, grouping of blast genomic aberrations into specific CNA-profiles can be operable on a BFM-based treatment backbone, clearly identifying distinct prognostic patient groups.

The major challenge was to demonstrate the CNA profile’s prognostic significance within the established risk groups of IR and HR patient groups. It is noteworthy that the majority of ALL recurrences are still observed in the large group of IR patients. In AIEOP-BFM ALL 2000 protocol, 69% of relapses occurred in IR patients, which exemplifies that, for a majority of patients with disease recurrence, the precedent treatment stratification strategy does not adequately and effectively relate with their actual risk of relapse [13,14]. Consequently, current stratification algorithms still need improvement to lead to a more precise early characterization of patients at true increased risk of relapse [14]. In this context, one of the major advantages of the UKALL-CNA classifier was the ability to subdivide the cytogenetic CYTO-IR cohort into subgroups with significantly different outcomes [25,26]. In our study, the proposed CNA risk index represents a simple, feasible and pragmatic approach to clarify this gray, not well-defined spectrum of IR patients: within the established IR-group, EFS was 100.0% for IR/GR-CNA-profile patients while for IR/PR-CNA-profile subjects EFS rate was inferior of only 60.0% (*p* < 0.001). Strengthening the above results, we report a relapse rate of 0.0% vs. 33.3% for the IR/GR-CNA-profile compared to the IR/PR-CNA-profile subgroup. The EFS survival rates for the proposed IR/PR-CNA profile subgroup (60.0%) are very similar to the ones noted in the conventional treatment protocol HR-group (67.6%), which suggests the ability to identify a subgroup of adverse prognosis patients within the IR treatment group that may benefit from early treatment intensification.

In an attempt to gain insight also into the group of high-risk patients, we demonstrated that EFS rates accounted for 88.2% vs. 55.6% (*p* = 0.047) for the HR/GR-CNA-profile vs. the HR/PR-CNA-profile subgroups, respectively. HR/GR-CNA-profile patients presented with low relapse rate (5.6%) while HR/PR-CNA-profile patients demonstrated the very increased relapse rate of 42.1%.

Of note, the EFS survival rates for the proposed HR/GR-CNA profile subgroup (88.2%) are very similar to the ones noted in the conventional IR-group (87.5%), after following HR type of treatment.

This is another interesting finding of the current study, further highlighting the fact that the HR-group is also heterogeneous, with at least a distinct subgroup of patients that may be eligible for treatment de-escalation and lessening of treatment-related toxicities that represent a major survival obstacle in their outcome and quality of life.

Last but not least, the question of MRD dependency and integration was one of the most important issues addressed and highlighted in our study. The extent to which the presence of specific genetic abnormalities influences the kinetics of disease clearance is not fully understood, and there is no consensus on the best method for integrating genomic and MRD data to stratify patients [46]. Gupta et al. [47] reported that the molecular genetic profile in BCR-ABL1-negative and B-other pediatric ALL can further refine outcome prediction, in addition to end-induction MRD detection. Additionally, O’Connor et al. [46] suggested the genotype-specific MRD interpretation in risk stratification and on top of that, the UKALL-CNA classifier was validated in a cohort of 3239 patients treated on MRD-adapted protocols [26]. In our study, the proposed CNA risk index retained clinical utility and prognostic significance among both MRD-positive (days 15 and 33 of induction) and end-of induction MRD-negative subgroups (day 33). Of most importance, at the end of induction, application of the diagnostic CNA-Risk classifier for the FC-MRD_d33_- (MRD_d33_ < 10^−4^) proved still to be important, with GR-CNA and PR-CNA patient subgroups having EFS of 97.2% vs. 72.7%, respectively (*p* = 0.004). Among FC-MRD_d33_- (MRD_d33_ < 10^−4^) patients, the relapse rates were 2.8% vs. 22.7% in the GR-CNA and PR-CNA-profile subgroups, respectively, demonstrating the additive stratification benefit of the CNA profile among IR MRD-negative patients. Last, in multivariate analysis, the proposed two-tier CNA profile was the most important prognostic factor for relapse, yielding a hazard ratio of 20.2 (95% Confidence Interval: 4.2–96.3, *p* < 0.001).

## 5. Conclusions

In conclusion, the current study indicates that application of the proposed CNA-profile classifier is feasible in BFM-based treatment protocols, adding prognostic value to all established prognostic markers. It is integrating well with MRD levels and further refines the already existing stratification system. The application of this CNA-profile classifier apart from being feasible, is of low cost and easily interpretable, ready for application before the end of induction.

Our results indicate that this novel genomic risk index can lead to early identification of distinct patient subgroups with different prognosis. Consequently, it can be incorporated in future risk-stratification algorithms, in an effort to further refine MRD-based stratification and improve treatment allocation algorithms and ultimate patient outcome. Naturally, due to the novelty of our proposed two-tier CNA risk index, applied in a limited patient cohort, the above significant indicative results need to be validated in future clinical trials, possibly within the frame of a new, multicentric, ALLIC BFM cohort, as planned, involving a much larger number of patients.

## Figures and Tables

**Table 1:** Baseline demographic, clinical, immunophenotypic, genetic and treatment characteristics of ALL patients evaluated by MLPA.

Characteristics	Number (%)
Total	85
Gender	
Male	48 (56.5)
Female	37 (43.5)
Immunophenotype	
B-ALL	77 (90.6)
Pro-B ALL	6 (7.1)
Common-B ALL	51 (60.0)
Pre-B ALL	20 (23.5)
T-ALL	8 (9.4)
ETP-ALL	2 (2.4)
Genetics	
ETV6/RUNX1	19 (22.3)
KMT2A rearrangements	3 (3.5)
BCR/ABL1	1 (1.2)
TCF3/PBX1	1 (1.2)
iAMP21	2 (2.3)
TCF3/HLF	0 (0)
Hyperdiploidy	18 (21.2)
Hypodiploidy	0 (0)
Treatment Protocol	
BFM 95/2000 modified	22 (25.9)
ALLIC BFM 2009	63 (74.1)
Protocol Risk Group	
Intermediate Risk	48 (56.5)
High Risk	37 (43.5)
FC-MRD Status	
FC-MRDd15 positive (MRD_d15_ ≥ 10^−4^)	73 (85.9)
FC-MRDd15 positive (MRD_d15_ ≥ 10^−3^)	66 (77.6)
FC-MRDd33 positive (MRD_d33_ ≥ 10^−4^)	26 (30.6)
Complete Remission (EOI-CR *)	
Yes	80 (94.1)
No	5 (5.9)
Allo-HSCT	17 (20.0)
Salvage Regimens #	15 (17.6)

FC-MRD: flow cytometry minimal residual disease, EOI-CR: end of induction complete remission, ETP: early T-precursor, allo-HSCT: allogeneic hematopoietic stem cell transplantation; * complete remission defined as BM morphological evaluation of <5% lymphoblasts by the end of induction; # salvage regimens include ALL REZ BFM 2002 Protocol, ALLIC Relapse Guidance 2016, Clofarabine/Cyclophosphamide/VP-16, Blinatumomab, Inotuzumab, allo-HSCT.

**Table 2:** Outcome characteristics of ALL patients stratified by CNA-profile.

Variable	Total (*n* = 85)		IR Group (*n* = 48)		HR Group (*n* = 37)	
	GR-CNA, n (%)	PR-CNA n (%)	GR-CNA n (%)	PR-CNA n (%)	GR-CNA n (%)	PR-CNA N (%)
Total *n* of patients Complete Remission *	51 (60.0)	34 (40.0)	33 (68.8)	15 (31.2)	18 (48.6)	19 (51.4)
	*n* (%)	*n* (%)	*n* (%)	*n* (%)	*n* (%)	*n* (%)
Yes	50 (98.0)	30 (88.2)	33 (100.0)	15(100.0)	17 (94.4)	15 (78.9)
No	1 (2.0)	4 (11.8)	0 (0.0)	0 (0.0)	1 (5.6)	4 (21.1)
Event						
Yes	3 (5.9)	15 (44.1)	0 (0.0)	6 (40.0)	3 (16.7)	9 (47.4)
No	48 (94.1)	19 (55.9)	33 (100.0)	9 (60.0)	15 (83.3)	10 (52.6)
Relapse						
Yes	1 (2.0)	12 (35.3)	0 (0.0)	5 (33.3)	1 (5.6)	8 (42.1)
No	50 (98.0)	22 (64.7)	33 (100.0)	10 (66.7)	17 (94.4)	11 (57.9)
Death						
Yes	3 (5.9)	8 (23.5)	0 (0.0)	1 (6.7)	3 (16.7)	7 (36.8)
No	48 (94.1)	26 (76.5)	33 (100.0)	14 (93.3)	15 (83.3)	12 (63.2)

* Complete remission defined as BM morphological evaluation of <5% lymphoblasts by the end of induction.

## Data Availability

Authors ensure that data shared are in accordance with consent provided by participants on the use of confidential data. The data presented in this study are available on request from the corresponding author. Hardcopies of all data and results are also available in patients’ files and collaborating involved laboratories. The data are not publicly available due to privacy and ethical restrictions.

## Abbreviations

ALL	Acute Lymphoblastic leukemia
CNA	Copy Number Alterations
FC	Flow Cytometry
MRD	Minimal Residual Disease
MRD_d15_	Minimal Residual Disease on day 15 of induction therapy
MRD_d33_	Minimal Residual Disease on day 33 of induction therapy
EOI	End of Induction
SR	Standard Risk
IR	Intermediate Risk
HR	High Risk
GR	Good Risk
PR	Poor Risk
MLPA	Multiple ligation probe amplification
OS	Overall Survival
EFS	Event-free survival
PCR	Polymerase chain reaction
BM	Bone marrow
